# PATTERNS OF SELF-EMPLOYMENT AND HEALTH TRAJECTORIES IN LATER LIFE: EVIDENCE FROM CHINA

**DOI:** 10.1093/geroni/igad104.0556

**Published:** 2023-12-21

**Authors:** Yu-Chih Chen

**Affiliations:** The University of Hong Kong, Hong Kong, Hong Kong

## Abstract

Self-employment is a major form of work among older adults, yet most research has focused on its antecedents, with a focus on developed countries. Additionally, the impact of self-employment on health and well-being remains understudied. This study extends the current literature by investigating self-employment in China, a country with a unique self-employment landscape encompassing both agricultural and non-agricultural types. Using data from the 2011-2018 China Health and Retirement Longitudinal Study, this study investigated the impacts of self-employment on physical, mental, and cognitive health among 5,492 older adults. Latent class analysis was used to identify self-employment patterns. Multilevel models were employed to assess the associations between self-employment patterns and health outcomes, adjusting for baseline health conditions. Results showed that three patterns of self-employment were identified: consistently self-employed, wage worker, and switchers who transitioned between self-employment and wage work. Compared to wage workers, self-employed individuals and switchers have lower self-rated health and life satisfaction. No significant differences were found in depressive symptoms and cognitive scores. Notably, such effects were stronger for agriculture self-employment than the non-agriculture self-employment. The study highlights that most older Chinese are pushed into self-employment, which can adversely affect their health outcomes. Structural efforts to support older adults to stay engaged at work, such as age-friendly job redesign, anti-ageism measures in the workplace, or pension reform, would promote health and economic well-being among older adults in China.

